# Berberine and Itraconazole Are not Synergistic *in Vitro* against *Aspergillus fumigatus* Isolated from Clinical Patients

**DOI:** 10.3390/molecules16119218

**Published:** 2011-11-03

**Authors:** Gao Lei, He Dan, Liu Jinhua, Yan Wei, Gao Song, Wang Li

**Affiliations:** 1 Department of Pathogenobiology, Norman Bethune College of Medicine, Jilin University, Jilin University Mycology Research Center (JUMRC), No.126 Xinmin Street, Changchun City, Jilin Province 130021, China; 2 Technical Center of Jilin Entry-Exit Inspection and Quarantine Bureau (JLCIQ) of China, Changchun City, Jilin Province 130062, China

**Keywords:** *Aspergillus fumigates*, berberine, itraconazole, ergosterol biosynthesis pathway

## Abstract

The incidence of *Aspergillus fumigatus* infections has become more frequent as a consequence of widespread immunosuppression. At present, the number of available antifungal agents in the clinic is limited, and most of them, such as itraconazole (ICZ), are toxic and show resistance. Berberine (BER) is a plant alkaloid used in the clinic mainly for alimentary infections. We have used BER and ICZ to measure *in vitro* resistance in *A. fumigatus* isolated from clinical patients. The minimum inhibitory concentration ranges of BER and ICZ were 4–256 and 0.031–0.250 μg/mL, respectively. In addition, against *A. fumigatus* IFM 40808 strain, the MIC_50_ values of BER and ICZ were 8 and 0.125 μg/mL. Using this strain, we compared the giant colonies with or without BER, and concluded that BER could restrain *A. fumigatus *mycelial growth and conidial pigment production. Combinations of the two drugs were also tested by the checkerboard assay to identify any functional interactions between them. Thirty-two out of 42 isolates had FICI values > 4.0, indicating that two drugs were mutually antagonistic. In conclusion, it is not advised that BER and ICZ be used in the clinic at the same time. Our results indicated that BER may inhibit *A. fumigatus* through the ergosterol biosynthesis pathway, like ICZ.

## 1. Introduction

**Figure 1:**
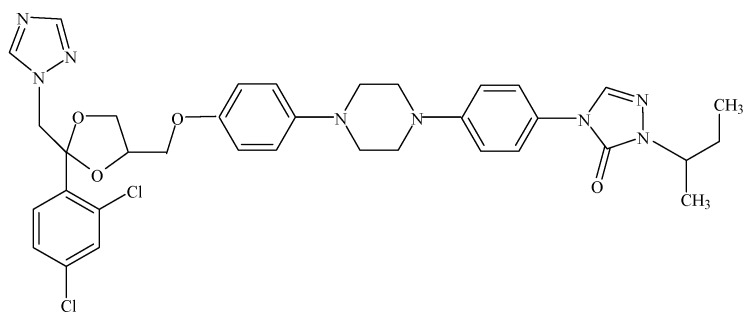
Structure of itraconazole.

*Aspergillus fumigatus* is an environmentally ubiquitous, spore-forming mold saprophyte. This airborne filamentous fungal pathogen is known to be a major cause of lethal lung infections in immunocompromised hosts and allergic asthma in atopic individuals [1]. Fungal infections including candidiasis, aspergillosis, cryptococcosis, and pneumocystosis often require hospitalization, with aspergillosis being the second most common cause [2]. Aspergillosis has high mortality rates, and its incidence has been increasing gradually [3]. Notwithstanding the increasing need for effective therapy, the range of antifungal agents available is limited, and some of the most effective agents are also toxic [4]. Treatment of *A. fumigatus* infections mainly involves azole derivatives, a class that includes itraconazole (C_35_H_38_Cl_2_N_8_O_4_, Figure 1), which has historically been the front-line drug in these treatments [5]. However, the pharmacological profile of azole drugs is determined and restricted by their liver toxicity, metabolic elimination, and pharmacokinetic drug-drug interactions involving CYP3A4 metabolic inhibition [6].

**Figure 2:**
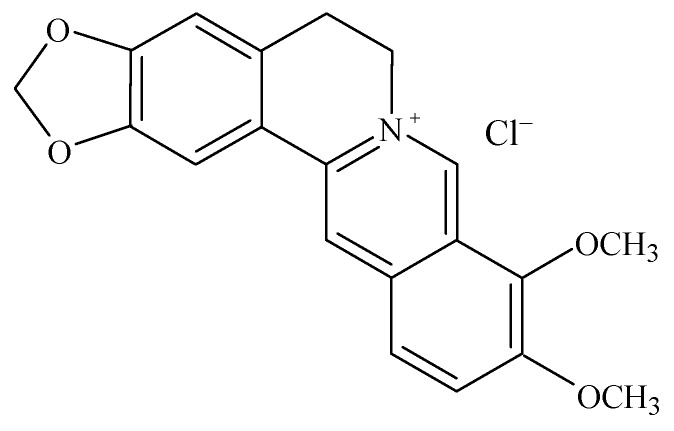
Structure of berberine.

Berberine ([C_20_H_17_ClNO_5_]^+^, Figure 2) is an isoquinoline plant alkaloid with a bright yellow color that is usually found in the stem bark, rhizomes and roots of the herb *Berberis * [7]. The alkaloid has multiple therapeutic actions and the use of berberine has been described for almost all disorders of the body. These plants are used medicinally in all traditional medical systems, and have a history of usage in Chinese and Korean medicine dating back at least 3,000 years. Berberine extract as a crude drug has been demonstrated to have significant antimicrobial activity against bacteria, viruses, protozoans, fungi, yeast [7,8], chlamydia and helminths (worms) [9]. The drug has been used in Chinese and Indian medicines for the treatment of bacterial diarrhea, intestinal parasitic infections, and ocular trachoma infections [10].

Testing of the effects of crude drugs on isolated fungi has been performed in China for over a thousand years as a means of identifying putative efficacious preparations. According to previous reports, berberine has both antifungal [4,11,12,13,14] and antibacterial effects [15,16,17,18] under *in vitro* conditions. We show here that berberine has antifungal effects on the growth of *A. fumigatus*, as determined by the minimum inhibitory concentration (MIC) method. We have also investigated whether berberine has a synergic effect with itraconazole *in vitro* when tested against *A. fumigatus* and whether the alkaloid berberine is a valid therapeutic agent against *A. fumigatus.* We have reached the conclusion that berberine and itraconazole inhibit *A. fumigatus* by similar mechanisms of action and are not synergistic.

## 2. Results and Discussion

### 2.1. Results

#### 2.1.1. Influence of BER and ICZ on *A. fumigatus*

The median MIC_50 _values of BER and ICZ for *A. fumigates* were individually calculated. The MIC range of BER over the 42 strains used was 4–256 μg/mL. The MIC range of ICZ (Table 1) was 0.031–0.250 μg/mL (42 strains). In addition, in *A. fumigatus* IFM 40808 cultures, the MIC_50_ of BER and ICZ was 8 and 0.125 μg/mL.

**Table 1:** Action of BER and ICZ alone (MIC_50_, μg/mL) or in combination (FICI value) against *A. fumigatus* isolates from clinical patients. Drug interactions were tested using the checkerboard microdilution method at a final inoculum of 0.5–2.5 × 10^3^ CFU/mL, using RPMI 1640 medium buffered with 0.165 M MOPS. Final drug concentrations ranged from 0.036–0.5 μg/mL for ICZ and 1–128 μg/mL for BER. Plates were incubated at 35 °C for 48 h prior to analysis. The fractional inhibitory concentration index (FICI) is the sum of the MIC of each drug in combination divided by the MIC of the drug used alone. A FICI value ≤0.5 is ‘synergy’; FICI > 4.0 is ‘antagonism’ and FICI > 0.5 and ≤4.0 is ‘no interaction’.

Strain No.	MIC_50_ (μg/mL)		FICI
	BER	ICZ	
IFM 40808	8	0.125	4.5
JLCC 30436	256	0.250	3.7
JLCC 30468	8	0.031	5.5
JLCC 30470	8	0.031	8.0
JLCC 30482	32	0.063	3.5
JLCC 30490	8	0.031	4.2
JLCC 30506	32	0.063	4.7
JLCC 30507	32	0.063	3.9
JLCC 30540	8	0.031	6.8
JLCC 30541	4	0.031	7.0
JLCC 30542	4	0.063	5.0
JLCC 30545	128	0.125	2.9
JLCC 30653	16	0.063	5.5
JLCC 30654	8	0.063	2.5
JLCC 30685	128	0.125	7.5
JLCC 30717	8	0.031	6.9
JLCC 30753	8	0.031	5.5
JLCC 30782	4	0.031	8.0
JLCC 30796	16	0.063	4.8
JLCC 30854	8	0.031	3.0
JLCC 30858	256	0.250	4.1
JLCC 30859	256	0.250	3.5
JLCC 30860	4	0.031	5.0
JLCC 30883	128	0.125	4.6
JLCC 30890	8	0.031	6.5
JLCC 31354	8	0.031	5.0
JLCC 31473	8	0.031	8.0
JLCC 31495	4	0.031	8.0
JLCC 31631	8	0.031	5.7
JLCC 31584	8	0.031	8.0
JLCC 31658	8	0.031	7.0
JLCC 31660	4	0.031	6.0
JLCC 31661	8	0.031	5.1
JLCC 31671	8	0.031	4.4
JLCC 31683	8	0.031	4.5
JLCC 31699	32	0.125	4.0
JLCC 31715	8	0.031	4.9
JLCC 32069	16	0.063	4.4
JLCC 32071	256	0.250	3.7
JLCC 32712	8	0.031	3.9
JLCC 33802	8	0.031	6.6
JLCC 33803	4	0.031	8.0

**Figure 3:**
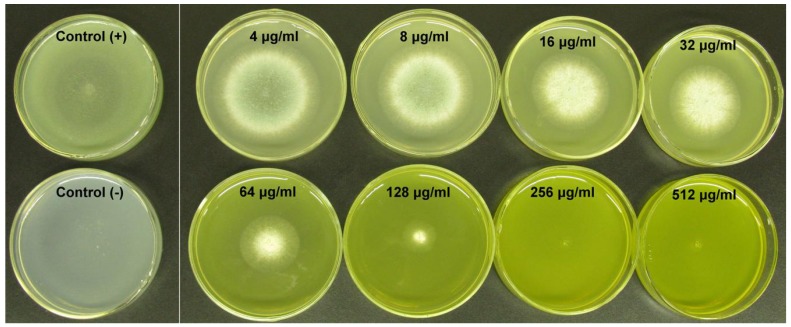
*A. fumigatus* spores were spotted onto the centre of PDA plates containing different concentrations of BER (512, 256, 128, 64, 32, 16, 8 and 4 μg/mL), and control (+) was treated with DMSO only (the final concentrations of DMSO less than 1%), control (−) was only a PDA without fungus. All plates were incubated at 35 °C. After 7 days, bioassays showed, with increasing concentrations of BER, it could significantly inhibit the growth of *A. fumigates*. The MIC was taken as the lowest concentration of the drug that showed not any fungal colonies growth on the agar plates. When the concentration was more than 256 μg/mL, there were no colonies visible on plates.

After 5 days the bioassays showed that with increasing concentrations of BER, the growth of *A. fumigates* could be significantly inhibited. When the concentration was more than 256 μg/mL, there was no colonies visible on plates (see Figure 3), and therefore we concluded that the MIC_50_ was 256 μg/mL on the agar plates.

**Figure 4:**
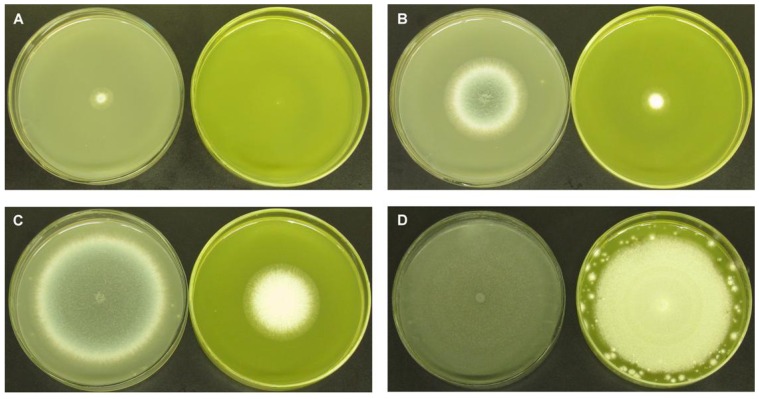
*A. fumigatus *without (left) and with (right) treatment with BER (at the 1/2 MIC_50_ 128 μg/mL), comparing giant colonies grown on PDA at 35 °C for 3 days (**A**), 5 days (**B**), 7 days (**C**) or 10 days (**D**). Effects of BER on colony size, mycelial growth and conidial pigment were investigated. Images shown are representative of three independent experiments.

**Figure 5:**
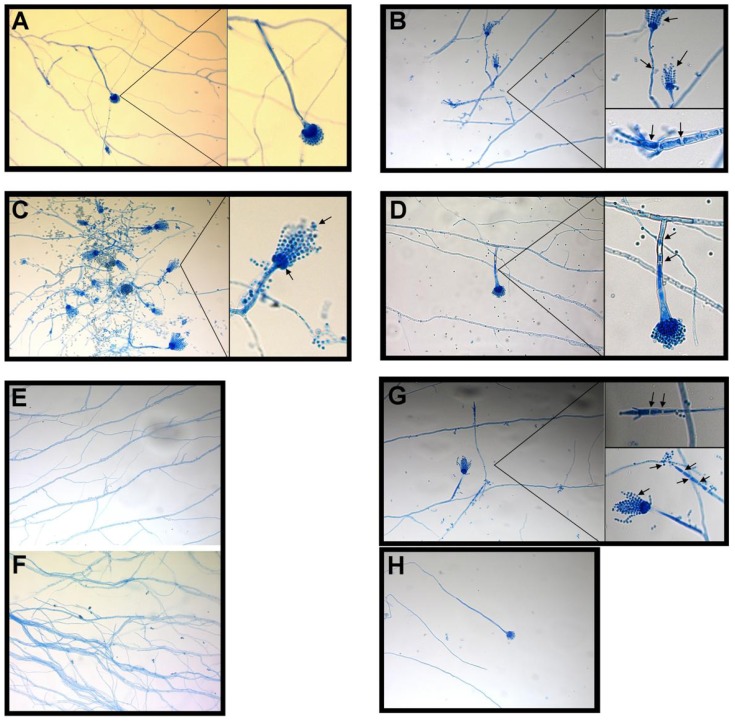
Lactophenol cotton blue preparation of a slide culture of *A. fumigatus* (original × 200). Images of intact structures of normal and mutation (treated with BER at 1/2 MIC_50_ on plates) strains of *A. fumigatus* using an Axiovert 200 MAT microscope (Zeiss, Germany) by slide culture techniques at 3 day (**A**). Microscopic view of *A. fumigatus* showing typical columnar, uniseriate conidial heads. *A. fumigatus* grows at 45 °C. Note the conical-shaped terminal vesicles supporting a single row of phialides on the upper two-thirds of the vesicle. Vesicles are uniseriate and are covered by phialides / conidia on only the distal half. Conidia arise in chains and tend to sweep toward the central axis (**B**, **C**, **G**). Strains treated with BER (at the 1/2 MIC_50_) showing the appearance of a septum in the conidiophores (**B**, **D**, **G**), elongation (**G**, **H**), and distortion of conidial heads and obscuration of metulae and phialides (**B**, **C**, **G**). Some mycelia were wreathed and some phialides arose circumferentially and were biseriate or uniseriate, with circumferential conidia obscuring vesicles (**E**, **F**).

We compared the giant colonies of *A. fumigatus* treated with DMSO alone and treated with BER (at the 1/2 MIC_50_ values of 128 μg/mL) to investigate the effect of BER on colony size (Table 2), mycelial growth and conidial pigment at 3, 5, 7 and 10 day. Colonies changed from granular to cottony, velvety, or powdery. At 3 day, the colonies were green, darkening to blue-green at 5 day, with a white apron at the colony margin. *A. fumigatus* on PDA at 7 days had a typical green-gray surface pigment with a suede-like surface texture consisting of a dense felt of conidiophores. *A. fumigatus* treated with BER exhibited smaller colony size, slower mycelial growth, and reduced conidia. These cultures also lost conidial pigment such that the conidial surface observed was white rather than green-gray (Figure 4). Figure 5 shows the results of morphological analysis of *A. fumigatus *in slide culture, with notable changes occurring in various morphological features (Figure 5B–H). These results demonstrate that BER can restrain *A. fumigatus *growth, development and conidial pigmentation.

**Table 2:** The colony sizes of *A. fumigatus* alone or *A. fumigatus* treated with BER (at 128 μg/mL).

Giant colonies	Diameter (cm)			
	3 d	5 d	7 d	10 d
*A. fumigatus* alone	1.19 ± 0.09	4.29 ± 0.11	6.99 ± 0.13	9.00 ± 0.10
*A. fumigatus* treated with BER	0.04 ± 0.07	1.50 ± 0.08	3.92 ± 0.09	7.10 ± 0.09

#### 2.1.2. Fractional Inhibitory Concentration Index

Calculation of FICI values for drug combinations is a useful indicator of the nature of functional drug interactions. FICI values between 0.5 and 4.0 indicate no interaction, whereas values below 0.5 and above 4.0 signify synergistic and antagonistic interactions, respectively. Table 1 shows FICI values for the BER-ICZ combination in each of the 42 *A. fumigatus* strains used in this study. All calculated values were above 0.5, demonstrating that the drug interaction was not synergistic in any strain. Furthermore, 32 of the 42 strains gave a FICI value greater than 4.0, suggesting that the interaction between BER and ICZ in these strains was mutually antagonistic (*i.e*., acting via identical pathways).

#### 2.1.3. Assessment of Ergosterol Content

**Figure 6:**
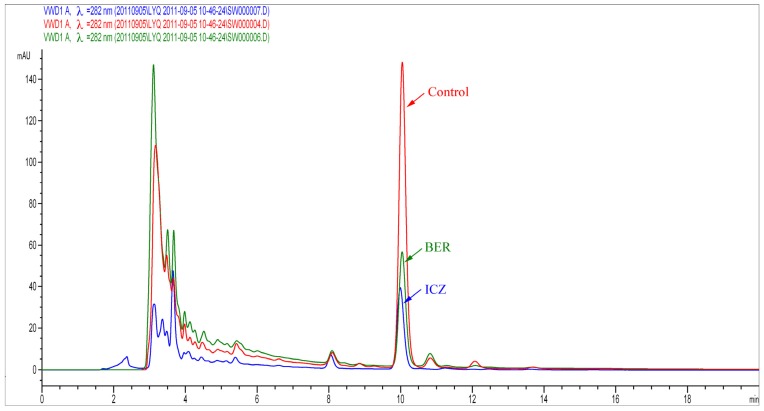
HPLC analysis of ergosterol in *A. fumigates*. Red was Control, green was BER, blue was ICZ.

The results indicated that the fungal dry weight was 254.40 ± 0.12 mg for the control group, 150.60 ± 0.14 mg for the BER group, and 97.49 ± 0.13 mg for the ICZ group. The HPLC results suggested that the retention time of ergosterol was about 10 min whatever the extraction process used and the chromatograms in this zone were very similar (Figure 6). The standard curve was linear over the range of ergosterol concentrations from 0.0001 to 0.01 μg/mL, *r*^2^ = 0.99983 with a slope of 25,924.25 and passing through the origin. The assay showed the variation coefficient less than 10%. The lower limit of the detector was established at detection 0.0001 μg/mL. Under our experimental conditions, ergosterol content in the control group was 0.067 mg/mL, in the BER group it was 0.025 mg/mL, and in the ICZ group it was 0.018 mg/mL. The results show ergosterol contents of BER and ICZ group were decreased (*P* < 0.01). 

#### 2.1.4. Real-Time PCR

**Figure 7:**
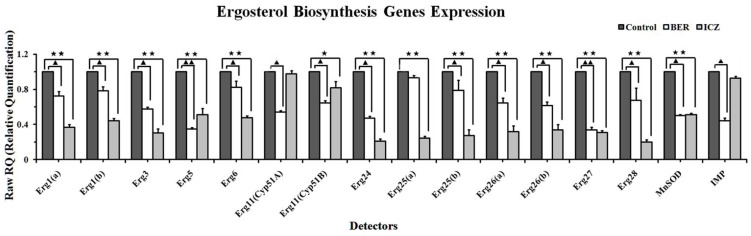
Comparison of the changes in expression of ergosterol biosynthesis pathway gene, determined by real-time PCR approaches. Student’s *t*-test was used to determine whether fold changes in gene expression induced by BER or induced by ICZ were different from untreated control. Significant differences are denoted with ^▲^ (*P*_BER_ < 0.05), ^★^ (*P*_ICZ_ < 0.05) and ^▲▲^ (*P*_BER_ < 0.01), ^★★^ (*P*_ICZ_ < 0.01). The fold expression in *A. fumigatus* treated with BER or ICZ as compared with that in untreated control. The calibrator was non-treatment, for which the fold expression was 1.0.

Many genes encoding proteins in the ergosterol biosynthesis pathway show differential expression levels following exposure to BER and ICZ. Seventeen representative target genes and a control gene (GAPDH) were chosen for validation with real-time PCR, and results are shown in Figure 7. Gene expression profiles with BER were similar to those with ICZ, reflecting that the actions of these two agents are largely similar. The *Erg5 *(Cytochrome P450 sterol C-22 desaturase), *Cyp51A* (14-alpha sterol demethylase *Cyp51A*), *Cyp51B *(14-alpha sterol demethylase *Cyp51B*)and *IMP* (Integral membrane protein) genes have been shown to be downregulated by medicines, and expression of these genes in *A. fumigatus *treated with BER was more significant than in *A. fumigatus *treated with ICZ (Fold-change value, *Erg5 *was 0.347 ± 0.018 *vs.* 0.509 ± 0.073, *P* < 0.05; *Cyp51A* was 0.540 ± 0.017 *vs.* 0.974 ± 0.042, *P* < 0.05; *Cyp51B* was 0.645 ± 0.029 *vs.* 0.819 ± 0.070, *P* < 0.05; and *IMP* was 0.439 ± 0.036 *vs.* 0.927 ± 0.022, *P* < 0.05). However, most other genes except *Erg27* (3-keto steroid reductase) and *MnSOD* (Mn superoxide dismutase) that were regulated by the BER and ICZ to similar degrees or were more strongly downregulated by ICZ (*P* < 0.05 or *P* < 0.01).

### 2.2. Discussion

BER is an alkaloid isolated from Chinese and Korean medicinal plants that notably inhibits the growth of a wide range of *Candida* species [19,20,21]. BER is clinically used in diseases such as urinary tract infections and conjunctivitis caused by bacteria like *Escherichia coli*, *Staphylococcus aureus* and *Shigella dysenteriae*. In recent years, research has shown that BER can also be used to treat fungal and viral infections and other diseases such as diabetes, hypertension, hyperlipidemia and arrhythmia [22,23,24,25,26,27,28]. Recently, investigators have used BER combined with amphotericin B to treat disseminated candidiasis in a mouse model, and others have investigated synergistic interactions of BER with fluconazole against fluconazole-resistant clinical isolates of *Candida albicans* [4,22,29,30]. Up to now, there has been no research on BER inhibition of *A. fumigatus*, or on the potential benefits of the combined effects of BER and ICZ. Our current study is the first to apply BER to clinically isolated *A. fumigatus *strains, and the first to use BER and ICZ in a combination treatment. Our results show that BER can inhibit the production of conidial pigment in *A. fumigatus* and consequently cause aberrations in *A. fumigatus* morphology and a reduction in its sporulation rate.

It is well known that drugs respond very differently in different background media. So we gave details as to how the MIC was obtained for BER on potato dextrose agar. We also show that BER and ICZ are mutually antagonistic in terms of their ability to inhibit *A. fumigatus* isolated from clinical patients. Furthermore, we have investigated the biomechanisms by which BER and ICZ inhibit the *A. fumigatus* ergosterol biosynthesis pathway. Ergosterol, one of the most basic components in fungal membranes, is involved in a multitude of biological functions, for instance, membrane fluidity regulation, distribution and activity of integral proteins and control of the cell cycle [31]. Indeed, controlling ergosterol and its biosynthetic pathway can impact fungal growth. Knowledge of the effects on the ergosterol biosynthesis in *A. fumigatus* has been key in the development of many antifungal drugs in clinical use and can hopefully aid the design of novel drugs [32]. Previous studies have shown that the majority of triazole drugs such as ICZ inhibit *A. fumigatus* isolates by targetingthe ergosterol biosynthesis pathway [33]. In the present experiments, we found BER can inhibit ergosterol like ICZ. The results from our gene expression experiments also indicate that BER significantly inhibits gene expression in the *A. fumigatus* ergosterol biosynthesis pathway and that BER is significantly more effective than ICZ at inhibiting expression of the *Erg5*, *Cyp51A*, *Cyp51B* and *IMP *genes, which are related to pigment production in *A. fumigatus* conidia. It could be that inhibition of these genes causes albino characteristics in *A. fumigatus *conidia. The *IMP* gene is closely related to cell wall biosynthesis [34] and, by inhibiting its expression, BER may thus inhibit biosynthesis of fungal cell walls and cause growth and developmental aberrations in *A. fumigates * [35].

## 3. Experimental Section

### 3.1. Strains, Media and Conditions

*A. fumigatus* strain IFM 40808 was isolated from the lung of a 54-year-old female Japanese patient with invasive aspergillosis, and provided from the Medical Mycology Research Center, Chiba University, Japan. The other *A. fumigatus* strains used in this study (41 strains: JLCC 30436, JLCC 30468, JLCC 30470, JLCC 30482, JLCC 30490, JLCC 30506, JLCC 30507, JLCC 30540, JLCC 30541, JLCC 30542, JLCC 30545, JLCC 30653, JLCC 30654, JLCC 30685, JLCC 30717, JLCC 30753, JLCC 30782, JLCC 30796, JLCC 30854, JLCC 30858, JLCC 30859, JLCC 30860, JLCC 30883, JLCC 30890, JLCC 31354, JLCC 31473, JLCC 31495, JLCC 31631, JLCC 31584, JLCC 31658, JLCC 31660, JLCC 31661, JLCC 31671, JLCC 31683, JLCC 31699, JLCC 31715, JLCC 32069, JLCC 32071, JLCC 32712, JLCC 33802, JLCC 33803) were isolated from 244 randomly selected clinical fungal infection patients at the Hospital of Jilin University between January 2008 and December 2010. Each strain was identified according to its physiological and morphological characteristics, and identified by molecular biology (mitochondrial cytochrome *b* gene, mt cyt *b*) [36]. All strains were preserved in the Culture Collection of Jilin University Mycology Research Center (JLCC), China.

Strains were cultured on potato dextrose agar medium (PDA; Becton Dickinson Co., Sparks, MD, USA) in C-shaped streaks at 35 °C for 3–4 days. Conidial suspensions were maintained in 0.9% NaCl-Tween 80 (0.01%) in sterile water at room temperature.

### 3.2. Antifungal Drug Susceptibility Testing

The minimum inhibitory concentration (MIC) of itraconazole (ICZ) (Sigma, Sigma-Aldrich Co., Louis, MO, USA) and berberine (BER) (Aldrich, Sigma-Aldrich Co., Louis, MO, USA) were tested following the Clinical and Laboratory Standards Institute document M38-A2 [37], with end points measured at 48 h (All drugs dissolved in dimethylsulfoxide (DMSO) (Sigma) and an initial ICZ concentration of 1,600 μg/mL，an initial BER concentration of 20,480 μg/mL). ICZ and BER were used over concentration ranges of 0.03–16 μg/mL and 1–512 μg/mL. *A. fumigatus* spores were harvested from the stock cultures and their concentration adjusted to 1.0 × 10^6^ colony-forming units (CFU)/mL by 0.9% NaCl-Tween80 sterile water. Then spores were spotted onto the centre of PDA plates containing different concentrations of BER (512, 256, 128, 64, 32, 16, 8 and 4 μg/mL，BER was dissolved in DMSO), and control group drug was used DMSO only (the final concentrations of DMSO less than 1%). The plates were incubated at 35 °C, and growth of filamentous fungi on plates was monitored after 7 days. The MIC_50_ was taken as the lowest concentration of the drug that showed not any fungal colonies growth on the agar plates [38].

### 3.3. Strain Culture

Giant colonies of *A. fumigatus* IFM 40808 treated without and with BER (at the 1/2 MIC_50_ of plate) were compared for size of colony, mycelial growth and conidial pigment at 3, 5, 7, 10 day. We also examined the intact structures of normal and BER strains at 3 day using an Axiovert 200 MAT microscope (Zeiss, Freiberg, Germany) using slide culture techniques described previously [39].

### 3.4. Fractional Inhibitory Concentration Index

Drug interactions were tested using the checkerboard microdilution method [40]. The checkerboard tests were performed by a broth microdilution reference procedure at a final inoculum of 1.0 × 10^6^–5.0 × 10^6^ CFU/mL, using RPMI 1640 medium (Roswell Park Memorial Institute, Sigma, Sigma-Aldrich Co., Louis, MO, USA) buffered with 0.165 M MOPS (3-(*N*-morpholino)propanesulfonic acid). Final concentrations ranged from 0.036 to 0.5 μg/mL for ICZ, and 1 to 128 μg/mL for BER. Ninety-six shadow masks were incubated at 35 °C for 48 h prior to analysis. The fractional inhibitory concentration index (FICI) is the sum of the MIC of each drug in combination divided by the MIC of the drug used alone. A FICI value ≤0.5 is classed as ‘synergy’, whereas a FICI value >4.0 denotes ‘antagonism’. Values >0.5 but ≤4.0 indicate that there is ‘no interaction’ between the drugs [41].

### 3.5. Assessment of Ergosterol Content

#### 3.5.1. Total Ergosterol Extraction

The well-developed *A. fumigatus* sample was cultured on a slant which was rinsed with 10 mL sterile water (0.9% NaCl-Tween80). Conidial suspension (10^6^ CFU/mL) was mixed with RPMI 1640 medium at 1:10. The media joined the MIC_50_ of BER and ICZ respectively (All drugs were dissolved in DMSO), and compared with control group (receiving DMSO only). The fungi were incubated at 35 °C, at 110 rpm shaking. *A. fumigatus* were grown separately in 150 mL flasks containing adequate medium. After 3 days, mycelia were collected by ﬁltration and washed with sterile phosphate-buffered saline (PBS, pH = 7.4), and then freeze-dried (LABCONCO FreeZone Triad Freeze Dry Systems, Kansas City, MO, USA) and weighted (fungal dry weight).

To a weight of dried mycelia (50 mg) was added 25% alcoholic KOH (potassium hydroxide solution) (3:2 methanol-ethanol, 25 mL) and then the mixture was homogenised by vortexing for 2 min. The mixed culture was incubated in a 90 °C water bath for 2.5 h. After cooling to room temperature, the saponified mixture was extracted in a fume hood with ether (20 mL), and vigorously vortexed for 15 min at room temperature. After 2.5 h the upper ether layer was transferred to a clean glass tube and evaporated with a centrifugal evaporator (EYELA, CVE-2000, Tokyo, Japan) at 20 °C. These dry residues were re-dissolved again in 1 mL of methanol and used for high performance liquid chromatography (HPLC) analysis.

Ergosterol standard (purity > 98%) was purchased from Sigma (Fluka, Buchs, Switzerland). A standard curve consisting of 0.0001, 0.0005, 0.001, 0.002, 0.005, 0.01 mg/mL. All stock solutions (the samples and standards) were filtered through 0.45-m-pore-size polytetraﬂuoroethylene membranes (Acrodisc, Waters, Waltham, MA, USA) [42].

#### 3.5.2. HPLC Analysis

Ergosterol contents were analyzed using an Agilent 1200 Series Rapid Resolution LC System (G1316A, Agilent Technologies, Santa Clara, CA, USA) including a -ZORBAX Extend C18 column (250 mm × 4.6 mm, 5 μm). Eluent: methanol/water (97/3) (100% HPLC grade). Flow Rate: 1 mL/min. Temperature: 25 ± 2 °C. Detection: UV (ultraviolet) at 282 nm. Each experiment were run triplicate and repeated at least three times.

### 3.6. Real-Time PCR

Using *A. fumigatus* IFM 40808, we compared the ergosterol biosynthesis pathway of *A. fumigatus* treated with either BER or ICZ (each at the MIC_50_ *i.e*., the MIC at which 50% of isolates are inhibited). Conidial suspensions were maintained at 1.0 × 10^6^ CFU/mL and inoculated in RPMI 1640 medium with DMSO, 1640 medium with BER (at the MIC_50_), 1640 medium with ICZ (at the MIC_50_). Cultures were incubated aerobically at 35 °C/110 rpm for 96 h, and examined daily before being collected and freeze-dried. Total RNA (messenger RNA) was prepared using the Qiagen RNeasy mini kit (Qiagen, Valencia, CA, USA) and VERSA Mini Nucleic Acid Extraction Workstation (Aurora Biomed, Vancouver, BC, Canada). The RNA was then reverse transcribed using the Quantitect Reverse Transcription Kit (Qiagen). The resultant cDNAs (complementary DNA) were subsequently analyzed by quantitative PCR (polymerase chain reaction) using a SYBR green master mix in an ABI 7500 thermocycler (Applied Biosystems, Foster City, CA, USA) following the manufacturer's recommended protocols. Table 3 shows the primers used in the real-time PCR experiments. Amplifications were performed with the following parameters: an initial preheat at 95 °C for 10 s, followed by 45 cycles at 95 °C for 5 s, 60 °C for 32 s, to detect and quantify the fluorescence at a temperature above the denaturation of primer-dimers. Once amplifications were completed, melting curves were obtained to identify PCR products. For each sample, PCR amplifications to quantify the expression of the constitutively-expressed GAPDH (Glyceraldehyde-3-phosphate dehydrogenase) gene were performed as a reference using the primer pair F-GAPDH-F + F-GAPDH-R (Table 3) [43]. The experiment was repeated five times. The expression of each tested gene in the BER-treated or ICZ-treated sample relative to that of untreated sample was calculated using the 2*^−ΔΔCt^* method [44,45,46].

**Table 3:** Primers used in real-time PCR analysis.

Locus tag	Protein description	Primer sequence (5′-3′)	Size of PCR product (bp)
AFUA_1G04720(*Erg1*)	C-8 sterol isomerase	F: TGCGATGGTCGACTTCATTGT	105
		R: ACCCATAGCACCACCAGCATT	
AFUA_5G07780(*Erg1*)	Squalenemonooxygenase *Erg1*	F: ATTGACAGGAGGCGGAATGAC	112
		R: GCTGCTTCATGACTCGCTTTG	
AFUA_6G05140(*Erg3*)	Sterol delta 5,6-desaturase	F: AAGCCGTTTGTGTACGAACCC	113
		R: AAAGAAGCTCAGGAACTGGCG	
AFUA_1G03950(*Erg5*)	Cytochrome P450 sterol C-22	F: TCAATTCGCCAGCTTACGTCA	101
	desaturase	R: ATTCAACGTGCTCCTTGCCA	
AFUA_4G03630(*Erg6*)	Sterol 24-c-methyltransferase	F: CGGATCGTTCAAGCACATGAC	107
		R: TTTCCATAGCTCCGCAGAACC	
AFUA_4G06890(*Erg11*)	14-alpha sterol demethylase *Cyp51A*	F: TGCGCACATGATGATAACCC	101
		R: TCGAGGACTTTTGGCTGTGAG	
AFUA_7G03740(*Erg11*)	14-alpha sterol demethylase *Cyp51B*	F: AAAATTCTCCGGCGTTCCAG	100
		R: TTTTCCTTGAAGCGAGCGC	
AFUA_1G03150(*Erg24*)	C-14sterol reductase	F:CGATTGGCTGATGTCATGGTC	102
		R:TATGGCCTGCTTCTGCATGTC	
AFUA_4G04820(*Erg25*)	C-4 methyl sterol oxidase *Erg25*	F:GCCGAATATGCATCGCCTATT	102
		R:TCGTGAAGATATGCAGGTCGC	
AFUA_8G02440(*Erg25*)	C-4 methyl sterol oxidase	F:AGGCGGATTTTGCCGTTCT	115
		R:GCAACCGTACCCATCAACACA	
AFUA_2G15030(*Erg26*)	C-3 sterol dehydrogenase/C-4 decarboxylase	F: AAGCATCCCCGACCGTTTT	112
		R: AAGAAGACGGTATGCGGCAAG	
AFUA_2G17400(*Erg26*)	C-3 sterol dehydrogenase/C-4 decarboxylase family protein	F:GGTGCAGATTGGCAACAACAA	102
		R: ATTTTGGCATGTAACCAGCGC	
AFUA_4G11500(*Erg27*)	3-keto steroid reductase	F:ACGAGTCACCTTTGTGGCTGA	105
		R:CACGATCGCATCGAGTTTAGG	
AFUA_2G11500(*Erg28*)	Ergosterol biosynthesis protein *Erg28*	F:CTGCCCAAATGGCTCGTCT	104
		R:TGGAGGCTGTTGATTGCAGAG	
AFUA_1G14550(*MnSOD*)	Mn superoxide dismutase	F: CCTACGTCAATGGCCTGAATG	100
		R: GAATTTGATCGCTTGCTGCA	
AFUA_6G07820(*IMP*)	Integral membrane protein	F: TGCATTTACCACATTGGCCA	105
		R: CAGAGCCACAATGATAGCCCA	
AFUA_5G01030(*GAPDH*)	Glyceraldehyde3-phosphate dehydrogenase	F: TCGCTGAATGCCAATTTCGT	106
		R: AGCATCGACACTGGCGATATG	

### 3.7. Statistical Analysis

Statistical analysis was performed with SPSS version 13.0 (SPSS Inc., Chicago, IL, USA). For all results analyses, Student’s *t *test was used. *P* < 0.05 was considered statistically significant. 

## 4. Conclusions

In conclusion, our study shows that while BER is effective in restraining the growth of several different *A. fumigatus* strains, it is not synergistic with ICZ against *A. fumigatus *isolated from clinical patients. Therefore, it is not advised that BER and ICZ be used at the same time in the clinic. This observation is the first report showing evidence of mutual antagonism between BER and ICZ. BER significantly inhibits gene expression in the ergosterol biosynthesis pathway of *A. fumigatus* like ICZ.
